# Screening ginseng saponins in progenitor cells identifies 20(R)-ginsenoside Rh_2_ as an enhancer of skeletal and cardiac muscle regeneration

**DOI:** 10.1038/s41598-020-61491-4

**Published:** 2020-03-18

**Authors:** Ah Ra Kim, Seon-Wook Kim, Ba-Wool Lee, Kuk-Hwa Kim, Woong-Hee Kim, Hong Seok, Ji-Hyung Lee, JungIn Um, Soon-Ho Yim, Youngkeun Ahn, Suk-Won Jin, Da-Woon Jung, Won Keun Oh, Darren R. Williams

**Affiliations:** 1 New Drug Targets Laboratory, School of Life Sciences, Gwangju Institute of Science and Technology, Gwangju, Jeollanam-do 61005 Republic of Korea; 2 Korea Bioactive Natural Material Bank, Research Institute of Pharmaceutical Sciences, College of Pharmacy, Seoul National University, Seoul, 08826 Republic of Korea; 3 Department of Pharmaceutical Engineering, Dongshin University, Naju, Jeollanam-do 58245 Republic of Korea; 4 Cell Regeneration Research Center, Department of Cardiology, Chonnam National University Hospital/Chonnam National University Medical School, Gwangju, 61469 Republic of Korea; 5 Developmental Genetics Laboratory, School of Life Sciences, Gwangju Institute of Science and Technology, Gwangju, Jeollanam-do 61005 Republic of Korea; 6 Yale Cardiovascular Research Center, Department of Internal Medicine, Yale University School of Medicine, New Haven, CT 06511 USA

**Keywords:** Cell biology, Biologics

## Abstract

Aging is associated with increased prevalence of skeletal and cardiac muscle disorders, such as sarcopenia and cardiac infarction. In this study, we constructed a compendium of purified ginsenoside compounds from *Panax ginseng* C.A. Meyer, which is a traditional Korean medicinal plant used to treat for muscle weakness. Skeletal muscle progenitor cell-based screening identified three compounds that enhance cell viability, of which 20(*R*)-ginsenoside Rh_2_ showed the most robust response. 20(*R*)-ginsenoside Rh_2_ increased viability in myoblasts and cardiomyocytes, but not fibroblasts or disease-related cells. The cellular mechanism was identified as downregulation of cyclin-dependent kinase inhibitor 1B (p27^Kip1^) via upregulation of Akt1/PKB phosphorylation at serine 473, with the orientation of the 20 carbon epimer being crucially important for biological activity. In zebrafish and mammalian models, 20(*R*)-ginsenoside Rh_2_ enhanced muscle cell proliferation and accelerated recovery from degeneration. Thus, we have identified 20(*R*)-ginsenoside Rh_2_ as a p27^Kip1^ inhibitor that may be developed as a natural therapeutic for muscle degeneration.

## Introduction

There is a long history of the use of *Panax ginseng* C.A. Meyer (*P. ginseng*) in Korean traditional medicine to treat reduced strength and cardiovascular disorders^1–3^. This medicinal plant was considered to be of high value in East Asia and has been associated with numerous therapeutic effects on the human body^3^. Detailed guidelines concerning the use of *P. ginseng* in Korean traditional medicine were recorded in the SohoDang miscellany of Taekyoung Kim (1850–1927) and Samjung-Yolam (1908)^4^. There has been much research effort to identify the active components that give *P. ginseng* its biological and pharmacological efficacy, but progress has fallen short of completely characterizing these compounds^3,5^.

The increasing lifespan of human society is linked with a greater prevalence of degenerative diseases^6,7^. For example, sarcopenia is the aging related degeneration of skeletal muscle mass (0.5–1% loss per year after the age of 50) that results from a deterioration in the proliferative capacity of resident muscle stem cells (termed satellite cells)^8^. Patients become progressively weaker, have greater propensity to fall over and may lose their ability to live independently. Aging is also a risk factor for cardiovascular diseases. For example, myocardial infarction (heart attack) is a leading cause of death, irrespective of race or ethnicity^9^. In contrast to skeletal muscle, cardiac muscle has little capacity for regeneration after degeneration^10^. Fibrous scar tissue is produced that can compromise heart function and lead to clinical heart failure^11^. Due to the historical use of *P. ginseng* to treat general weakness and disorders of the circulation^1–3^, we investigated whether it was possible to isolate an active compound that mediated these therapeutic effects. A library of 39 purified ginsenosides was screened in skeletal muscle progenitor cells (myoblasts) to detect compounds that increased cell proliferation, which is an indicator of the potential to enhance muscle regeneration^12^. 20(R)-ginsenoside Rh_2_ (CPP531) was identified as an enhancer of myoblast proliferation. Further experiments showed that CPP531 treatment improved recovery in animal models of skeletal and cardiac muscle degeneration. CPP531 treatment *in vitro* increased Akt1/PKB activation and repressed expression of cyclin-dependent kinase inhibitor 1B (p27^Kip1^).

## Results

### Ginsenoside compendium screening identified 20(*R*)-ginsenoside Rh_2_ as an enhancer of skeletal myoblast proliferation

**Figure 1:**
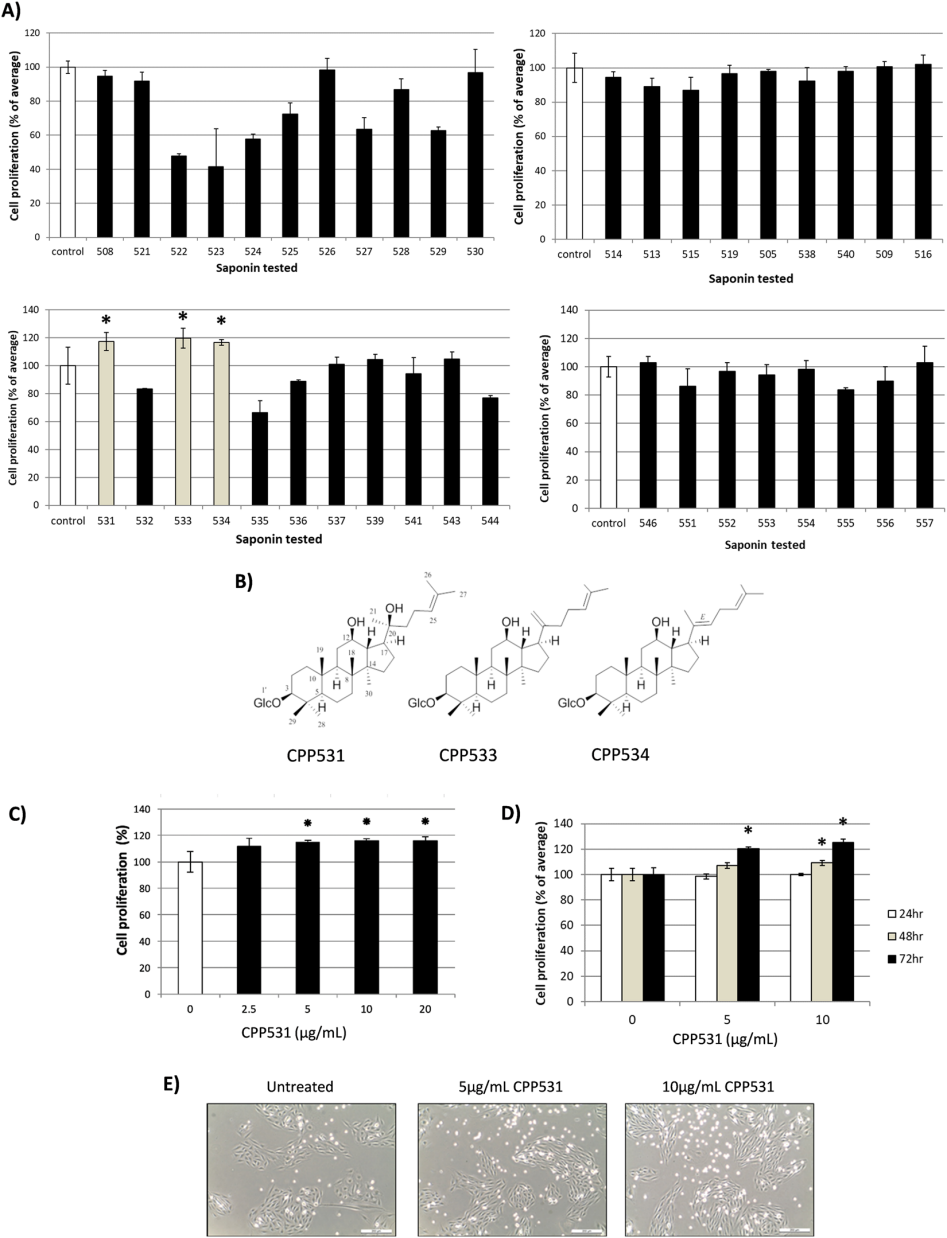
(**A**) Screening result of a compendium of 39 ginsenosides from *P. ginseng* for enhancers of myoblast proliferation. C2C12 myoblasts were treated with 5 µg/mL of each ginsenoside for 72 h. (**B**) Structure of the ′hit′ ginsenosides CPP531, ginsenoside Rk_2_ (designated as CPP533) and isoginsenoside Rh_3_ (designated as CPP534). (**C**) MTT assay showing the dose-dependent effect of CPP531 on C2C12 myoblast proliferation. Myoblasts were treated with CPP531 for 72 h. (**D**) MTT assay showing the time-dependent effect CPP531 treatment on C2C12 proliferation. (E) Micrographs of C2C12 myoblasts treated with CPP531 for 72 h. Scale bar = 200 µm. For (**A–D**): *p < 0.05 for significantly increased proliferation compared to vehicle treated myoblasts.

Previous research has shown that compounds which increase the proliferation of skeletal muscle progenitor cells have the potential to be developed as therapeutics for muscle wasting diseases^12^. A compendium of 39 ginsenosides purified from *P. ginseng* was screened in C2C12 murine myoblasts (Fig. 1A). Three ginsenosides were identified as ‘hits’ for increasing myoblast proliferation (compendium designations ‘CPP531’ for 20(*R*)-ginsenoside Rh_2_ and ‘CPP533’ for ginsenoside Rk_2_ and ‘CPP534’ for isoginsenoside Rh_3_) (Fig. 1B). ^1^H and ^13^C NMR data for these active compounds are provided in Table S1. Retesting these ‘hit’ ginsenosides indicated that only 20(*R*)-ginsenoside Rh_2_ (CPP531) produced a concentration-dependent effect on proliferation (Supplementary Fig. 1). CPP531 increased myoblast proliferation in a concentration and time-dependent manner (Fig. 1C,D). Microscopic observation of the myoblasts also indicated that CPP531 treatment increased cell numbers (Fig. 1E). The WST-1 assay is used as an alternative to the MTT assay, because the water-soluble formazan is produced outside of the cells^13^. The capacity of CPP531 to enhance myoblast proliferation was also demonstrated using this assay (Supplementary Fig. S2) and by flow cytometry analysis (Supplementary Fig. S3).

### CPP531 selectively increased proliferation in skeletal myoblasts compared to cancer cells and fibroblasts

**Figure 2:**
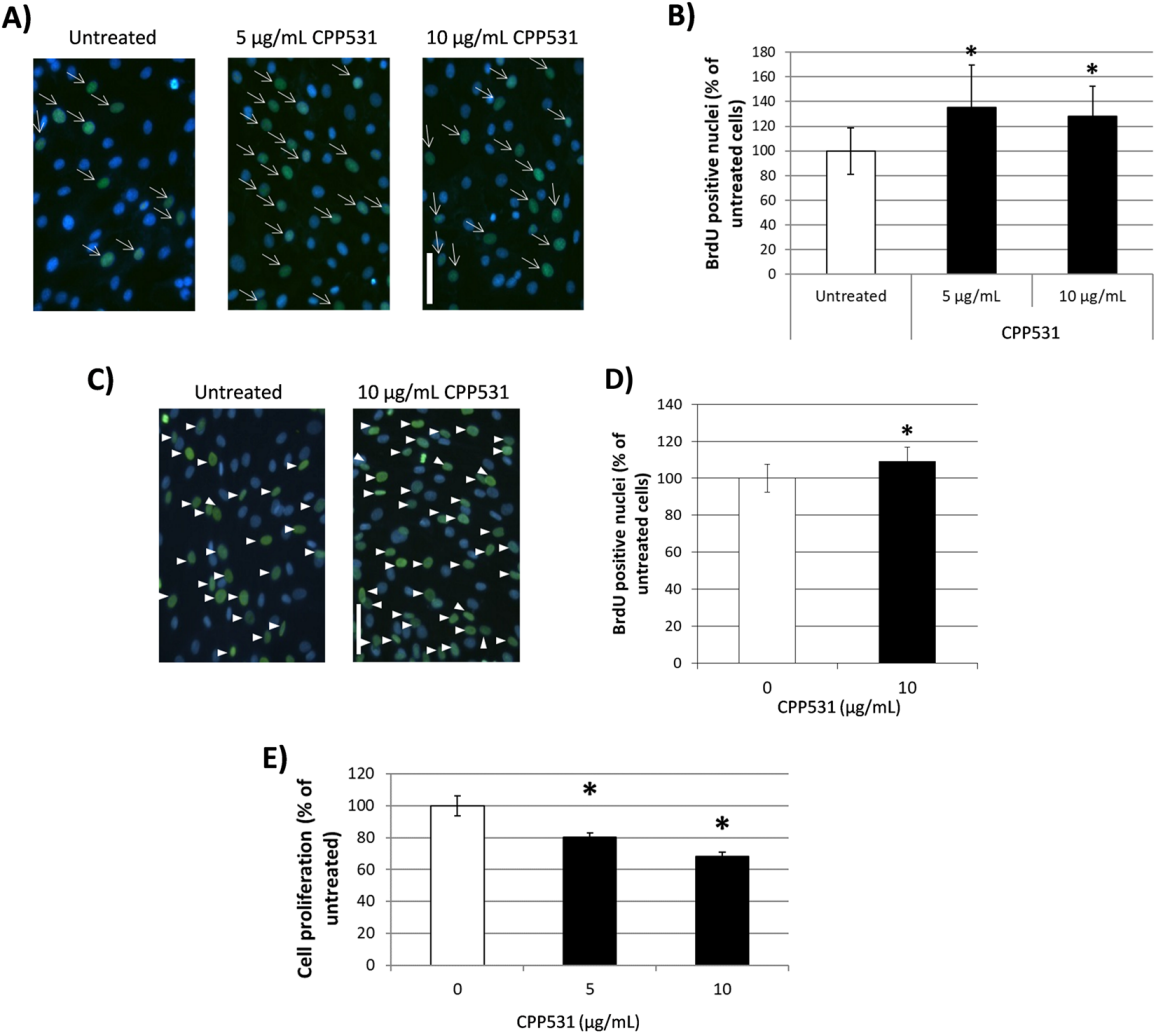
(**A**) BrdU immunostaining of C2C12 myoblasts treated with CPP531 for 72 h. Blue nuclei = DAPI, green nuclei = BrdU staining. Scale bar = 50 µm. (**B**) Quantification of BrdU labelled C2C12 nuclei. (**C**) BrdU immunostaining of human primary myoblasts treated with CPP531 for 72 h. Blue nuclei = DAPI, green nuclei = BrdU staining. Scale bar = 50 µm. (**D**) Quantification of BrdU labelled human myoblast nuclei. (**E**) Effect of 5 µg/mL CPP531 treatment for 72 h on proliferation in rat primary cardiac fibroblasts. For (**B**,**D**): *p < 0.05 for significantly increased BrdU nuclear staining compared to vehicle treated myoblasts; for (**E**) *p < 0.05 for significantly reduced proliferation compared to vehicle treated fibroblasts.

To further confirm that CPP531 increases proliferation in myoblasts, treated cells were immuno-stained for BrdU incorporation. It was observed that CPP531 increased BrdU incorporation into the nuclei of murine myoblasts (Fig. 2A,B). To assess if CPP531 is also effective in human myoblasts, primary myoblasts were purified from a biopsy taken from the gluteus maximus muscle of a male (29 years old) during knee surgery. Human myoblasts treated with 10 µg/mL CPP531 also increased proliferation (Fig. 2C,D; MTT assay data is provided in Supplementary Fig. 4).

To determine if the effect of CPP531 on cell proliferation is restricted to myoblasts or can occur in other cell types, especially cancer cells, various cell lines were treated with CPP531 and proliferation was measured using the MTT assay. YD-10B human oral carcinoma cells, HeLa human cervical carcinoma cells, HCT116 human colon carcinoma and NIH/3T3 fibroblasts did not show any significant increase in proliferation after CPP531 treatment (data not shown). Certain types of activated fibroblasts (termed myofibroblasts) contribute to degenerative diseases via enhanced proliferation^14^. Examples include cancer-associated fibroblasts (CAF) and cardiac fibroblasts (CdF)^15,16^. Treatment with CPP531 deceased the proliferation of CdF (Fig. 2E) and showed no significant effect on CAF proliferation (data not shown).

### CPP531 treatment in myoblasts activates Akt/PKB signaling and suppresses expression of p27^Kip1^ and p57^KIP2^

**Figure 3:**
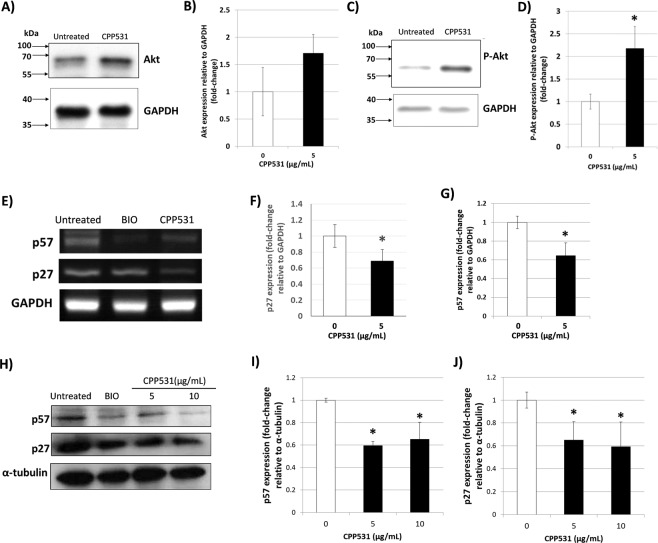
(**A**) Western blotting analysis of Akt expression in C2C12 myoblasts after treatment with 5 µg/mL CPP531 for 72 h. GAPDH was used as a loading control. (**B**) Quantification of Akt expression. (**C**) Western blotting analysis of phosphorylated Akt (P-Akt) expression in C2C12 myoblasts after treatment with 5 µg/mL CPP531 for 72 h. (**D**) Quantification of P-Akt expression. (**E**) RT-PCR analysis of p27^Kip1^ and p57^Kip2^ mRNA expression in C2C12 myoblasts after treatment with 5 µg/mL CPP531 or 5 μM BIO for 72 h. (**F**,**G**) Quantification of p27^Kip1^ and p57^Kip2^ mRNA expression. (**H**) Western blotting analysis of p27^Kip1^ and p57^Kip2^ expression in C2C12 myoblasts after treatment with 5 or 10 µg/mL CPP531, or 5 μM BIO, for 72 h. (**I**,**J**) Quantification of p27^Kip^ and p57^Kip2^ protein expression. For (**D**): *p < 0.05 for significantly increased expression compared to vehicle treated myoblasts. For (**F**,**G**) and (**I**,**J**): *p < 0.05 for significantly decreased expression compared to vehicle treated myoblasts.

To determine the mechanism by which CPP531 enhances myoblast proliferation, we measured activation of the Akt/PKB pathway, which is a major regulator of cell proliferation in response to external stimuli^17^. Using western blotting analysis, CPP531 treatment increased the expression of Akt, although this increase did not reach statistical significance (Fig. 3A,B). Western blotting indicated that phosphorylated Akt was significantly increased in myoblasts treated with CPP531 (Fig. 3C,D). Cell proliferation is negatively regulated by cyclin-dependent kinases inhibitors (CDKIs)^18^, which are, in turn, regulated by Akt activation^19,20^. RT-PCR analysis showed that CPP531 treatment decreased the expression of the CDKIs, p27^Kip1^ and p57^Kip2^ (Fig. 3E–G). For comparison, myoblasts were treated with 5 μM 6-bromoindirubin-3′-oxime (BIO), a compound previously shown to activate proliferation in refractory muscle cells and downregulate CDKI expression^21^. The negative effect of CPP531 treatment on p27^Kip1^ and p57^Kip2^ expression in myoblasts was confirmed by western blotting (Fig. 3H–J).

### CPP531 treatment inhibits p27^Kip1^ expression in cardiomyocytes and increases proliferation *in vitro* and *in vivo*

**Figure 4:**
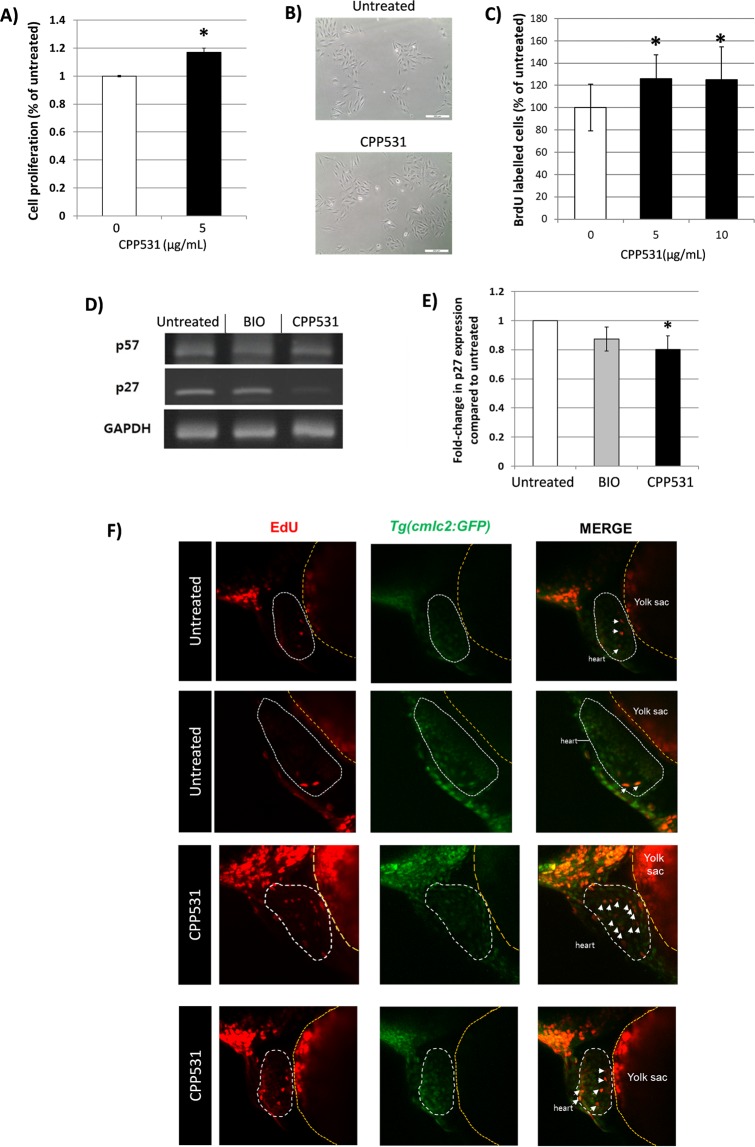
(**A**) MTT assay for cell proliferation in H9C2 cardiomyoblasts treated with 5 µg/mL CPP531 for 72 h. (**B**) Representative micrographs of the treated cardiomyoblasts. Scale bar = 200 μm. (**C**) BrdU labelling of rat neonatal cardiomyocyte nuclei after treatment with 5 µg/mL CPP531 5 µM BIO for 72 h. (**D**) RT-PCR analysis of p27^Kip1^ and p57^Kip2^ mRNA expression in rat neonatal cardiomyocytes after treatment with 5 µg/mL CPP531 or 5 μM BIO for 72 h. (**E**) Quantification of p27^Kip^ expression. (**F**) Effect of 5 µg/mL CPP531 on cardiomyocyte proliferation in *Tg(cmlc2:GFP)* transgenic zebrafish. 20 hpf larvae were treated with CPP531 until 48 hpf and EdU staining was carried out at 72 hpf. Heart tissue and neighboring yolk sac are designated with dashed lines. White arrows designate double-labelled, proliferating cardiomyocytes. Two representative fish from the CPP531 treated and untreated groups are shown. For (**A**): *p < 0.05 for significantly increased proliferation compared to vehicle treated cardiomyoblasts. For (**C**): *p < 0.05 for significantly increased BrdU labelling compared to vehicle treated cardiomyocytes. For (**C**): *p < 0.05 for significantly decreased expression compared to vehicle treated cardiomyocytes.

Considering the effect of CPP531 on myoblast proliferation, the effect of ginsenoside on proliferation was assessed in another muscle cell type, cardiomyocytes. As a first test, H9C2 cardiomyoblasts, which have been used to study drug effects in the heart^22,23^, were treated with CPP531 and proliferation measured using the MTT assay. CPP531 increased proliferation (Fig. 4A,B). To confirm this positive effect on cardiomyocyte proliferation, primary rat cardiomyocytes were treated with CPP531 and proliferation measured by BrdU staining. CPP531 increased proliferation in the primary cardiomyocytes (Fig. 4C). RT-PCR analysis indicated that CPP531 treatment decreased the expression of p27^Kip1^, but not p57^Kip2^, in the cardiomyocytes (Fig. 4D,E). The compound BIO compound was used as a positive control for the RT-PCR analysis, because it was previously shown to enhance cardiomyocyte proliferation and downregulate the expression of p27 ^Kip1^ ^24,25^. However, we could not detect significant p27 ^Kip1^ downregulation by BIO in our analysis.

To determine whether CPP531 treatment enhances cardiomyocyte proliferation *in vivo*, the zebrafish larval system was used, which allows direct visualization of dividing cardiomyocytes^25,26^. 20 hpf *Tg(cmlc2:GFP)* transgenic zebrafish larvae, which produce fluorescence from cardiomyocytes, were treated with 5 µg/mL CPP531 until 48 hpf. EdU staining and confocal microscopy assessment indicated that CPP531 treatment enhanced cardiomyocyte proliferation in the zebrafish (Fig. 4F).

### CPP531 increases cardiac recovery after myocardial infarction

**Figure 5:**
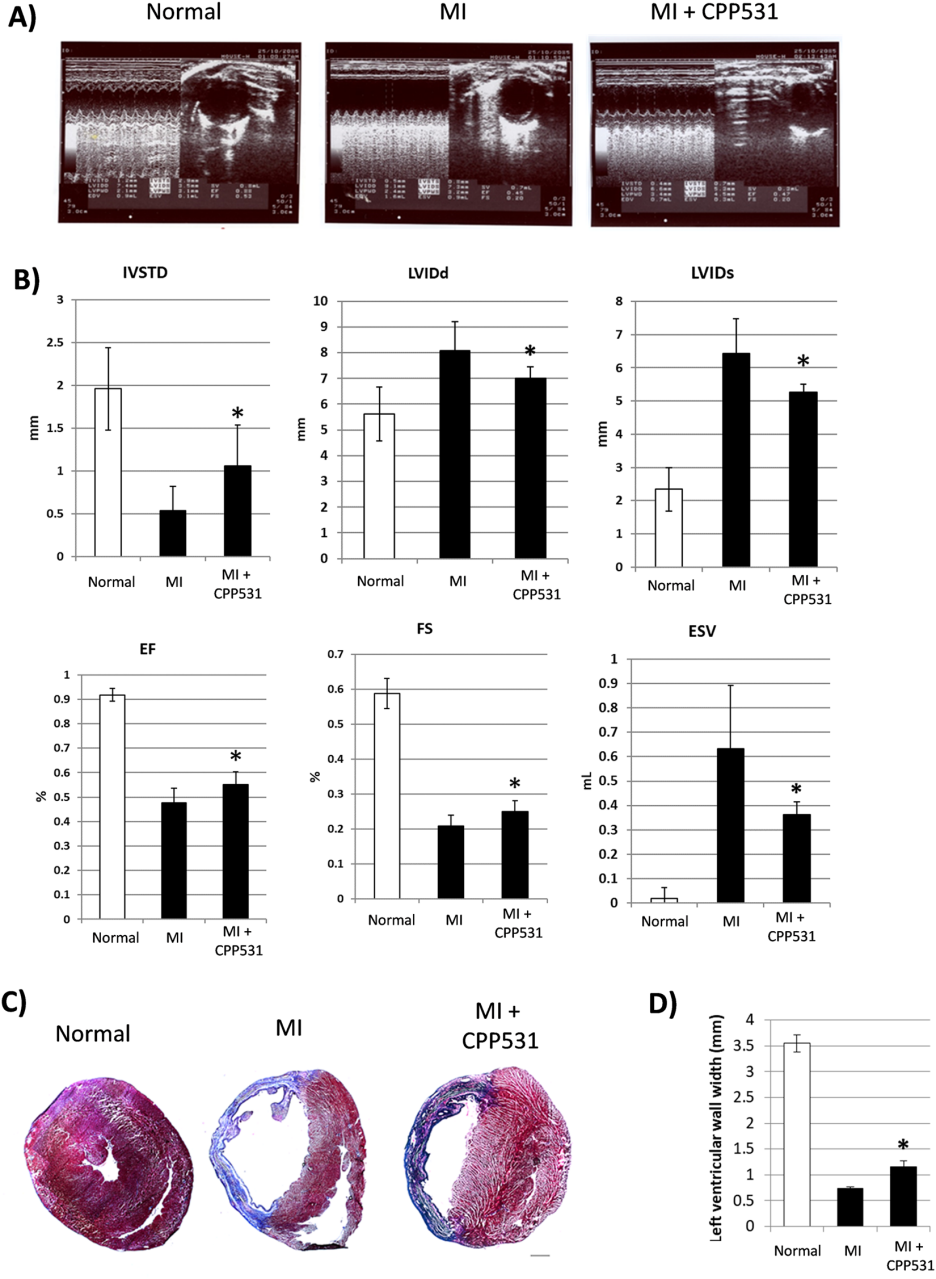
(**A**) Representative echocardiograms from rats with sham MI (designated as ‘Normal’), MI and 7 d vehicle treatment (designated as ‘MI’) or MI and 7 d treatment with CPP531 (designated as ‘CPP531’). (**B**) Effect of CPP531 treatment on cardiac function related parameters. IVSTD: interventricular septal end diastole, LVIDd: left ventricular internal dimension, diastole, LVIDs: left ventricular internal dimension, systole, EF: ejection fraction, FS: fractional shortening and ESV: end systolic volume. n = 8 rats per treatment group; *p < 0.05 for significant improvement compared to vehicle treated rats with MI. (**D**) Masson’s trichrome staining of representative rat hearts. Red staining indicates cardiomyocytes and blue staining indicates fibrotic scar tissue. Scale bar = 1 mm. (**E**) Effect of CPP531 treatment on left ventricular wall thickness. *p < 0.05 for significantly greater ventricular wall thickness compared to vehicle treated rats with MI.

The rat model of myocardial infarction (MI) was used to assess the effect of CPP531 on degenerated cardiac tissue. Functional recovery of the heart muscle was measured using echocardiography. It was observed that CPP531 treatment improved cardiac output parameters (intraventricular septal width in diastole, left ventricular internal dimension in diastole, left ventricular internal dimension in systole, ejection fraction, fractional shortening and end-systolic volume) (Fig. 5A,B). CPP531 treatment increased the presence of cardiomyocytes in the scar tissue and improved left ventricular thickness, which is an indicator of improved recovery^27^ (Fig. 5C,D). Immunostaining for cardiac troponin T and the Ki-67 marker of cell proliferation indicated a higher level of cardiomyocyte proliferation in the infarcted left ventricle of rats treated with CPP531 (Supplementary Fig. 5).

### CPP531 enhances skeletal muscle regeneration after injury

**Figure 6:**
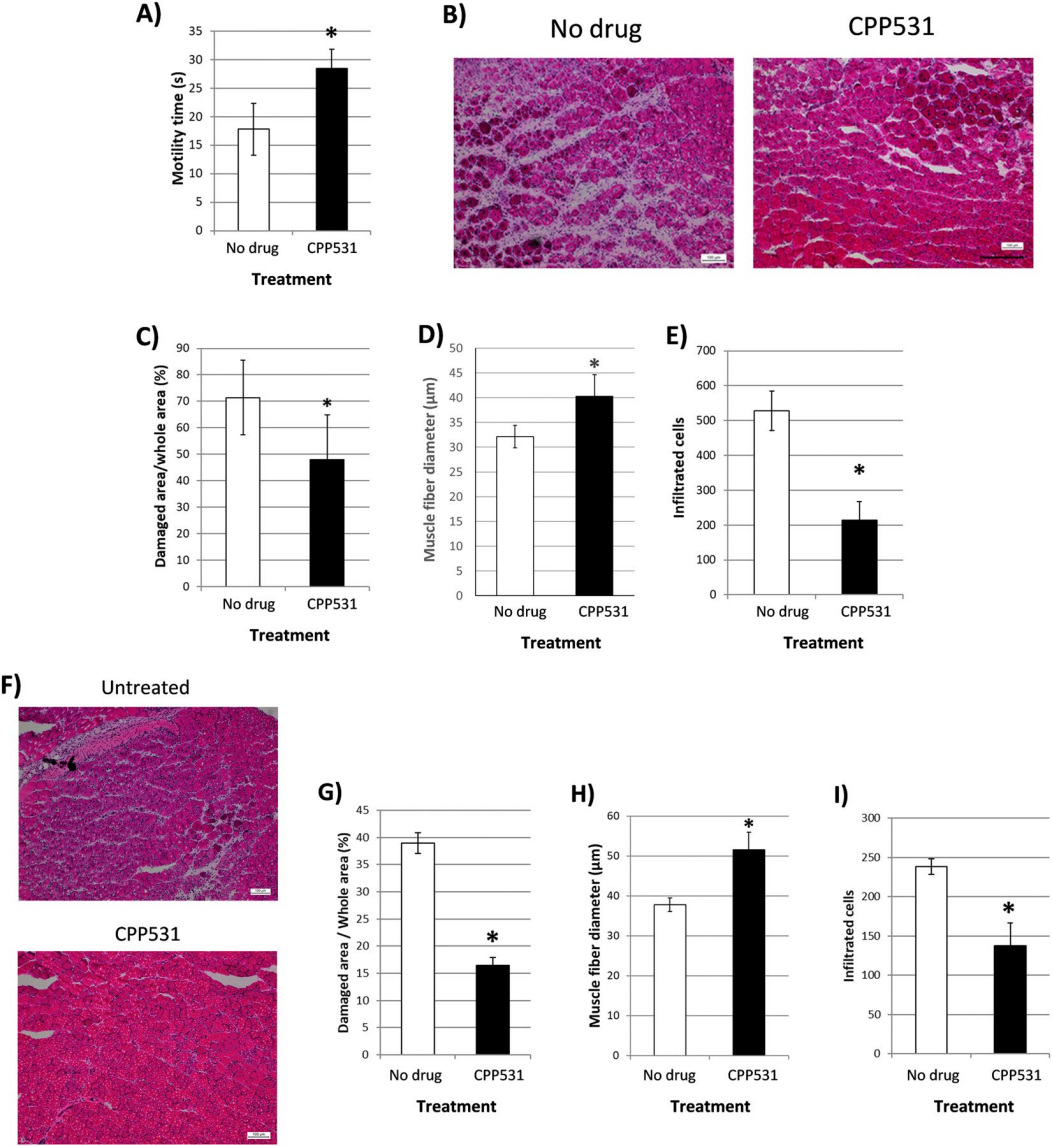
(**A**) Effect of CPP531 treatment on swim test motility time. *p < 0.05 for significant higher motility time compared to vehicle treated mice (designated as ‘no drug’). (**B**) Representative images of gastrocnemius muscle inflammation and fiber regeneration (indicated by the presence of small, centrally nucleated fibers) in vehicle treated mice and CPP531 treated mice 7 d after barium chloride injection. Scale bar = 100 µm. (**C**) Percentage of damaged gastrocnemius muscle tissue after 7 days. *p < 0.05 for significantly reduced damaged tissue compared to vehicle treated mice. (**D**) Muscle fiber diameter in vehicle treated mice and CPP531 treated mice after 7 d. *p < 0.05 for significantly larger diameter compared to vehicle treated mice. (**E**) Inflammatory cell count in the gastrocnemius muscle of untreated mice and CPP531 treated mice after 7 d. *p < 0.05 for significantly reduced inflammatory cell count compared to vehicle treated mice. (**F**) Representative images of the gastrocnemius muscle of untreated and CPP531 treated mice 14 d after barium chloride injection. Scale bar = 100 µm. (**G**) Percentage of damaged gastrocnemius muscle tissue after 14 d. *p < 0.05 for significantly reduced damaged tissue compared to vehicle treated mice. (**H**) Muscle fiber diameter in untreated mice and CPP531 treated mice at 14 d. *p < 0.05 for significantly increased diameter compared to vehicle treated mice. (**I**) Inflammatory cell count in the gastrocnemius muscle of untreated mice and CPP531 treated mice at 14 d. *p < 0.05 for significantly lower inflammatory cell count compared to the vehicle treated mice. n = 5 mice per treatment group.

To assess whether CPP531 treatment improves recovery in skeletal muscles after fiber degeneration, the intramuscular barium chloride model was employed. A forced swim exercise test was used to measure muscle performance 7d after barium chloride treatment. CPP531 treatment increased swimming motility time (Fig. 6A). Hematoxylin and eosin staining, and quantification of Masson’s trichrome staining, of the dissected gastrocnemius muscle showed reduced scarring in the CPP531 treated group (Fig. 6B,C). An increased proportion of smaller diameter fibers is a feature of regeneration after muscle injury, which then increase in diameter as the muscle heals^28^. One week after injury, mean fiber diameter was higher in the gastrocnemius muscle of CPP531 treated mice (Fig. 6D). Sites of muscle degeneration are associated with areas of inflammatory cell infiltration, which resolves as the muscle regenerates^29^. CPP531 treated mice showed reduced numbers of infiltrating cells (Fig. 6E). After 2 weeks of CPP531 treatment, the gastrocnemius muscle showed improved histology, reduced tissue degeneration, higher mean fiber diameter, and fewer inflammatory cells score compared to vehicle-treated mice (Fig. 6F–I).

## Discussion

In this study, we investigated the active components of *P. ginseng* leaf extract, which is used in Korean traditional medicine to treat muscle weakness and enhance blood circulation^1–3^. Screening a compendium of ginseng saponins identified the ginsenoside CPP531 as an inducer of myoblast proliferation that enhances both skeletal and cardiac muscle regeneration *in vivo*.

The screening system used in this study is based on an increase of myoblast proliferation, which has been used to identify compounds with therapeutic potential for treating skeletal muscle degeneration^12^. This screen detected CPP531 (designated as cpp531 in the compendium) as an enhancer of proliferation, but not the epimer, 20(S)-ginsenoside Rh_2_ (designated as CPP532; Supplementary Fig. S6). Ginsenoside Rk_2_, designated as CPP533, was also detected as a ‘hit’ in this screen (Fig. 1A,B). This result indicates that the orientation of the 20 carbon epimer is of critical importance for this biological activity.

White spots could be observed in the C2C12 myoblast cultures treated with CPP531 (Fig. 1E). We believe that the white spots in the cultures are myoblasts that have detached from the culture plate during the process of cell division and become refractile. Although there are more white spots in the 10 µg/mL treatment group compared to the 5 µg/mL group, our opinion is that the adherent cell number is increased after compound treatment, but does not reach statistical significance between the treatment groups. This is based on the MTT assay (Fig. 1C), which does not include the contribution from floating cells. We believe that this is why we could not detect significant increases in cell proliferation when comparing the 10 µg/mL and 5 µg/mL treatment groups.

Compared to CPP532, there are relatively few publications reporting the biological activities of CPP531. Kang, *et al*, showed that CPP531 produced antiviral properties and inhibited murine gammaherpesvirus infection^30^. Dong and Pang demonstrated that CPP531 enhanced bone formation in osteoblast precursor cells^31^ and Lui, *et al*, reported CPP531 as an inhibitor on osteoclastgenesis and bone resorption^32^. It was also shown that CPP531 and CPP532 can prevent photo-aging in keratinocytes, albeit by different mechanisms^33^, and both ginsenosides have been reported as anti-cancer compounds^34–36^. However, to our knowledge, the results presented herein are the first demonstration that CPP531 is a natural compound that increases myoblast and cardiomyocyte proliferation, and enhances skeletal and cardiac muscle regeneration *in vivo*. The therapeutic effects of CPP531 were verified using animal models of acute muscle damage; induction of myocardial infarction or injection of barium chloride (a skeletal muscle myotoxin). To further investigate the potential of CPP531 to treat muscle diseases, it may be interesting to test this small molecule in models of chronic muscle disease, such as the dexamethasone-induced mouse model of skeletal muscle wasting^37^ or the mouse hypertension model^38^. Additionally, the potential application of CPP531 as a drug for genetic muscle wasting disorders, such as Duchenne muscular dystrophy, could be assessed in the mouse *mdx* model^39^.

Ginsenoside compounds are known to regulate the expression and activity of numerous signaling receptors and ion channels, such as receptor tyrosine kinases, serotonin receptors, N-methyl-D-aspartate receptors and nicotinic acetylcholine receptors^40^. Our data shows that CPP531 treatment increases the activity of Akt/PKB, which is a major ‘hub’ for transducing signals from activated receptors (Fig. 3A–D). CPP531 treatment downregulated the expression of the CDKIs p27^Kip1^ and p57^Kip2^ in myoblasts, and p27^Kip1^ in cardiomyocytes, which is in accordance with previous studies of Akt/PKB, p27^Kip1^ and p57^Kip2^ ^18–20^. The compound BIO was previously shown to enhance proliferation and downregulate the expression of p27^Kip1^ in cardiomyocytes^24,25^. However, we could not detect significant p27^Kip1^ downregulation by BIO in our RT-PCR analysis (Figs. 3E and 4D). Recently, Dong and Pang reported that a potential target mechanism for CPP531 in osteoblast precursor cells is upregulated expression of the long non-coding RNA H19 (lncRNA H19)^31^. Future studies could assess link between this lncRNA and the activation of Akt/PKB signaling observed in our study to completely elucidate the mechanism of CPP531 in myoblasts and cardiomyocytes.

Skeletal muscle has been called “the last undrugged organ system”^41^. There is also a clinical need for new compounds that enhance cardiac muscle regeneration after myocardial infarction^42^. Previous studies have implicated ginsenosides in aspects of muscle function, such as glucose uptake^43^. The results presented in this manuscript demonstrate for the first time that a purified ginsenoside small molecule, CPP531, enhances myoblast and cardiomyocyte proliferation in both skeletal and cardiac muscle regeneration *in vivo*. Thus, CPP531 can be considered as a new candidate for further development as a natural-based therapeutic for degenerative diseases affecting the heart or skeletal musculature.

## Materials and Methods

### Reagents and antibodies

Barium chloride and collagenase type 2 solution (210 U/mL), were purchased from Sigma-Aldrich, St. Louis, USA. Pancreatin solution (0.6 mg/mL) was purchased from Thermo Fisher Scientific, Waltham, USA. An antibody for Akt (catalogue number #9272) was purchased from Cell Signaling Technology, Inc., Danvers, USA. Antibodies for Ser 473 phosphorylated Akt1 (catalogue number sc-514032), an antibody BrdU (catalogue number sc-32323) p27^Kip1^ (catalogue number sc-1641) and p57^Kip2^ (catalogue number sc-56341) were purchased from Santa Cruz Biotechnology, Dallas, USA. BIO ((2′Z,3′E)-6-Bromoindirubin-3′-oxime) was kindly provided by Professor Yong-Chul Kim, Laboratory of Drug Discovery, Gwangju Institute of Science and Technology, Korea.

### Cell culture

HCT116 human colorectal carcinoma cells, HeLa human cervical cancer cells, H9C2 murine cardiomyoblasts and NIH/3T3 murine fibroblasts, were purchased from the Korean Cell Line Bank. C2C12 murine myoblasts were purchased from Koram Biotech Corp., Seoul, Korea. Each cell type was cultured in Dulbecco’s minimum essential medium (DMEM) supplemented with 10% fetal bovine serum (FBS), 50 units/mL penicillin and 50 µg/mL streptomycin (PenStrep).

YD-10B human oral squamous cell carcinoma (OSCC) cells were purchased from the Korean Cell Line Bank. Seoul, Korea. CAF4 OSCC-associated fibroblasts were a generous gift from Professor Jin Kim, Yonsei University, Korea. YD-10B and CAF4 cells were cultured in DMEM and F-12 media (3:1 ratio) supplemented with 10% FBS and PenStrep.

Cardiomyocytes and cardiac fibroblasts were purified from 2 d-old Sprague-Dawley rats. In brief, the heart ventricles were dissected from the euthanized rats and washed in 4 °C PBS, sliced into small pieces with a scalpel and digested in 0.1% collagenase type 2 solution and 0.6 mg/mL pancreatin for 30 min with mild stirring. The supernatant was collected and centrifuged through a Percoll gradient (Sigma-Aldrich) at 1000 rpm for 5 min. The cardiac cell layer was harvested and cultured with DMEM supplemented with 10% FBS for 1 h. The non-adherent cardiomyocytes were then removed from the adherent cardiac fibroblasts and used for experiments. The proliferation culture for cardiac fibroblasts was DMEM supplemented with 10% FBS and 1% PenStrep. The fibroblasts were used for experiments at passage 2 or passage 3.

Human primary skeletal muscle myoblasts were isolated from a muscle biopsy taken from the gluteus maximus muscle of a male (29 years old) during knee surgery (IRB no. CNUH-2014-042; Chonnam National University Medical School, Korea). The experimental protocols were approved by the Institutional Review Board of Chonnam National University Medical School, Korea. All methods were carried out in accordance with the relevant guidelines and regulations of the Institutional Review Board of Chonnam National University Medical School, Korea, and informed consent was obtained from the participant.

The muscle biopsy was placed in sterile tube containing autoclaved PBS and 1% PenStrep. The connective tissue was dissected using a stereo microscope. The muscle tissue was then sliced into 1–2 cm pieces and each piece was transferred to a 10 cm tissue culture dish containing primary cell growth media (DMEM supplemented with 20% FBS, 10 µg/ml bovine insulin, 25 ng/mL human fibroblast growth factor, 10 ng/ml human epidermal growth factor and 1% PenStrep) for 5 days. Floating muscle tissues were removed and the adherent cells were sub-cultured every 2 d. The trypsinized cells were placed in a culture dish for 15 min, allowing sufficient time for the fibroblasts to attach. The media with floating myoblasts was harvested and transferred to another 10 cm culture dish. This process was repeated five times to remove contaminating fibroblasts.

For cell-based experiments, stocks of CPP531 were prepared at 2.5, 5, 10 and 20 mg/mL in the DMSO vehicle to keep the final concentration of DMSO at 0.1% for cell treatment.

### Ginsenoside library screening

A compendium of 39 ginsenosides was assembled by Professor Won Keun Oh, Department of Pharmacy, Seoul National University, Korea, as described below and listed in Table 1. C2C12 myoblasts were seeded in 96 well plates at a density of 1 × 10^3^ cells/well. 24 h later, the culture media was replenished and cells were treated with 5 µg/mL test ginsenoside for 72 h. Each ginsenoside was treated in triplicate. The effect of screened ginsenosides on myoblast proliferation was assessed using the MTT assay.

**Table 1:** Compendium of purified ginsenosides from *P. ginseng*.

No.	CPP No.	Saponin	Formula	Molecular mass (Da)^*^	Reference
1	505	20(*R*)-ginsenoside Rh_1_	C_36_H_62_O_9_	638.87	^47^
2	508	20(*S*)-ginsenoside Rg_2_	C_42_H_72_O_13_	785.01	^47^
3	509	20(*R*)-ginsenoside Rg_2_	C_42_H_72_O_13_	785.01	^48^
4	513	ginsenoside F_3_	C_41_H_70_O_13_	770.98	^49^
5	514	20(*E*)-ginsenoside F_4_	C_42_H_70_O_12_	767.00	^50^
6	515	ginsenoside F_5_	C_41_H_70_O_13_	770.98	^49^
7	516	20(*S*)-ginsenoside Rg_1_	C_42_H_72_O_14_	801.01	^47^
8	519	ginsenoside Re	C_48_H_82_O_18_	947.15	^47^
9	521	ginsenoside Rg_5_	C_42_H_70_O_12_	767.00	^51^
10	522	compound Y	C_41_H_70_O_12_	754.98	^52^
11	523	20(*S*)-protopanaxadiol	C_30_H_52_O_3_	460.73	^53^
12	524	3-deoxy-3-oxo-20(*S*)-protopanaxatriol	C_30_H_50_O_4_	474.71	^54^
13	525	dehydroprotopanaxatriol I	C_30_H_50_O_3_	458.71	^55^
14	526	dehydroprotopanaxatriol II	C_30_H_50_O_3_	458.71	^55^
15	527	compound K	C_36_H_62_O_8_	622.87	^56^
16	528	ginsenoside Rg_6_	C_42_H_70_O_12_	767.00	^57^
17	529	compound Mc	C_41_H_70_O_12_	754.98	^52^
18	530	20(*R*)-protopanaxadiol	C_30_H_52_O_3_	460.73	^53^
19	531	20(*R*)-ginsenoside Rh_2_	C_36_H_62_O_8_	622.87	^48^
20	532	20(*S*)-ginsenoside Rh_2_	C_36_H_62_O_8_	622.87	^48^
21	533	ginsenoside Rk_2_	C_36_H_60_O_7_	604.86	^58^
22	534	isoginsenoside Rh_3_	C_36_H_60_O_7_	604.86	^51^
23	535	gypenoside IX	C_47_H_80_O_17_	917.12	^59^
24	536	gypenoside XVII	C_48_H_82_O_18_	947.15	^60^
25	537	ginsenoside Rb_1_	C_54_H_92_O_23_	1109.29	^61^
26	538	ginsenoside Rb_2_	C_53_H_90_O_22_	1079.26	^61^
27	539	ginsenoside Rb_3_	C_53_H_90_O_22_	1079.26	^60^
28	540	ginsenoside Rc	C_53_H_90_O_22_	1079.26	^61^
29	541	ginsenoside Rh_4_	C_36_H_60_O_8_	620.85	^47^
30	543	bipinnatifidusoside F_1_	C_48_H_82_O_19_	963.15	^62^
31	544	notoginsenoside Fe	C_47_H_80_O_17_	917.12	^63^
32	546	vinaginsenoside R_4_	C_48_H_82_O_19_	963.15	^62^
33	551	3*β*,6*α*,12*β*-trihydroxy-27-nordammar-(*E*,*E*)- 20(22),23-diene-25-one	C_29_H_46_O_4_	458.67	^64^
34	552	ginsenoside Re_4_	C_47_H_80_O_18_	933.12	^65^
35	553	ginsenoside Rh_18_	C_48_H_80_O_18_	945.13	^66^
36	554	3*β*,20(*S*)-dihydroxydammar-24-en-12*β*,23*β*-epoxy-20-*O*-*β*-d-glucopyranoside	C_36_H_60_O_8_	620.85	^64^
37	555	6*α*,20(*S*)-dihydroxydammar-24-ene-3,12-dione	C_30_H_48_O_4_	472.70	^67^
38	556	6*α*,20(*S*),24(*S*)-trihydroxydammar-25-ene-3,12-dione	C_30_H_48_O_5_	488.70	^67^
39	557	6*α*,20(*S*),25-trihydroxydammar-23-ene-3,12-dione	C_30_H_48_O_5_	488.70	^67^

^*^The molecular masses are based upon the latest atomic mass data from the International Union of Pure and Applied Chemistry (IUPAC).

### MTT cell proliferation assay

Cell proliferation was measured using the MTT (3-(4,5-dimethylthiazol-2-yl)-2,5-diphenyltetrazolium bromide) assay, as previously described^44–46^. After compound incubation, the culture media was changed to 1X MTT solution (0.5 mg/mL in PBS) and the plate was incubated at 37 °C for 2 h. The MTT solution was carefully discarded. 80 µL DMSO was added to each well and the MTT absorbance was read at 540 nm using a microplate reader (VersaMax, Molecular Devices, USA).

### Isolation and characterization of ginsenosides from *P. ginseng*

Extensive studies about *P. ginseng* resulted in the isolation of 39 dammarane triterpenes and established as a compendium of Korean ginseng saponins. By comparison with previously reported spectroscopic information including NMR data, their structures were determined and identified as followed (Table 1). A Gilson high-performance liquid chromatography (HPLC) purification system (Gilson Inc., WI, USA) was used at a flow of 2 mL/min and UV detection at 205 and 254 nm with an Optima Pak C_18_ column (10 × 250 mm, 10 *μ*m particle size; RS Tech, Seoul, Korea)

The dried leaves of *P. ginseng* were purchased from Kumsan Ginseng Town, Chungcheongnam-do, Korea and authenticated by Professor W. K. Oh at Seoul National University. A voucher specimen (SNU2010-10) was deposited in the herbarium of the college of Pharmacy, at Seoul National University, Korea.

The dried leaves of *P. ginseng* (10 kg) were extracted with 80% EtOH (3 times, for 2 h each) using a sonicator. The combined extract was concentrated by an evaporator to yield a dried residue (850 g). Dried extract was suspended in 40% EtOH and then subjected to Diaion HP-20 macroporous resin (Mitsubishi Chemical Corp., Tokyo, Japan) open column chromatography (CC) (11 × 55 cm) and eluted with 40% EtOH, EtOH, and washed with acetone. Partial EtOH fraction was set aside for production of sapogenin by a series of chemical reaction. To obtain saponin rich fraction, remained EtOH and acetone fractions were combined (245 g) and subjected to silica gel CC (particle size: 63−200 *μ*m, Zeochem, Lake Zurich, Switzerland) (20 × 60 cm) and eluted with gradient system of EtOAc/MeOH from 10:1 to 0:1 to yield five fractions (F.1 – F.8).

F.1 (10.7 g) was subjected seilica gel CC with solvent system of CHCl_3_/MeOH to afford eight subfractions (F.1.1 – F.1.8). F.1.4 was preprocessed by reversed-phase CC (particle size: 75 *μ*m, nacalai tesque, Kyoto, Japan) with gradient system from 25% to 100% MeOH to give twenty two subfractions. F.1.4.13 was subjected to semi-preparative (semi-prep) HPLC with a MeOH/H_2_O (v/v, 76/24), resulting in the isolation of 6*α*,20(*S*)-dihydroxydammar-24-ene-3,12-dione (3.0 mg). F.1.4.15 was subjected to semi-prep HPLC with a MeOH/H_2_O (v/v, 78/22), resulting in the isolation of 3-deoxy-3-oxo-20(*S*)-protopanaxatriol (3.8 mg). F.1.6 was subjected to reversed-phase CC with gradient system from 33% to 100% MeOH to give nine subfractions. F.1.6.9 was subjected to semi-prep HPLC with a MeOH/H_2_O (v/v, 75/25), resulting in the isolation of 6*α*,20(*S*),25-trihydroxydammar-23-ene-3,12-dione (2.8 mg). F.1.8 was subjected to semi-prep HPLC with a MeOH/H_2_O (v/v, 80/20), resulting in the isolation of 6*α*,20(*S*),24(*S*)-trihydroxydammar-25-ene-3,12-dione (2.0 mg).

F.2 (15.3 g) was subjected to seilica gel CC with solvent system of CHCl_3_/MeOH to afford eight subfractions (F.2.1 – F.2.8). F.2.5 was subjected to reversed-phase CC with gradient system from 25% to 100% MeOH to give ten subfractions. F.2.5.8 was subjected to semi-prep HPLC with a MeOH/H_2_O (v/v, 70/30), resulting in the isolation of 3*β*,6*α*,12*β*-trihydroxy-27-nordammar-(*E*,*E*)- 20(22),23-diene-25-one (5.6 mg). Ginsenoside Rk_2_ (24.3 mg) and isoginsenoside Rh_3_ (24.4 mg) were isolated from F.2.5.10. 3*β*,20(*S*)-dihydroxydammar-24-en-12*β*,23*β*-epoxy-20-*O*-*β*-d-glucopyranoside (3.8 mg) was purified from F.2.6.7. F.2.7 was subjected to reversed-phase CC with gradient system from 25% to 100% MeOH to give fourteen subfractions. F.2.7.8 was subjected to semi-prep HPLC with a MeOH/H_2_O (v/v, 68/32), resulting in the isolation of 20(*R*)-ginsenoside Rh_1_ (10.5 mg). F.2.7.14.5 was subjected to semi-prep HPLC with a MeCN/H_2_O (v/v, 58/42), resulting in the isolation of compound K (2.8 mg), 20(*S*)-ginsenoside Rh_2_ (5 mg) and 20(*R*)-ginsenoside Rh_2_ (9.2 mg).

F.3 (30.5 g) was subjected to seilica gel CC with solvent system of CHCl_3_/MeOH (v/v, 40/1 to 0:1) to afford ten subfractions (F.3.1 – F.3.10). F.3.8 was subjected to semi-prep HPLC with a MeCN/H_2_O (v/v, 55/45), resulting in the isolation of ginsenoside Rh_4_ (6.3 mg). F.3.9 was subjected to reversed-phase CC with gradient system from 25% to 100% MeOH to give twenty-one subfractions. F.3.9.9 was subjected to semi-prep HPLC with a MeOH/H_2_O (v/v, 66/34), resulting in the isolation of ginsenoside F_5_ (35.0 mg). ginsenoside Rg_6_ (200.4 mg) and compound Mc (27.1 mg) were isolated from F.3.9.13 and F.3.9.21, respectively.

F.4 (70 g) was subjected to seilica gel CC with solvent system of CHCl_3_/MeOH (v/v, 20/0 to 0:1) to afford fifteen subfractions (F.4.1 – F.4.15). F.4.11 was subjected to reversed-phase CC with gradient system from 25% to 100% MeOH to give thirty-two subfractions. F.4.11.32 was subjected to reversed-phase MPLC with gradient system from 33% to 100% MeOH to give eight subfractions. compound Y (12.0 mg) and ginsenoside Rg_5_ (24.3 mg) were isolated from F.4.11.32.7.

F.5 (30.5 g) was subjected to Sephadex LH-20 (GE Healthcare, Little Chalfont, UK) (7 × 30 cm) with 100% MeOH to obtain seven subfractions (F.5.1 – F5.7). F.5.3 was subjected to reversed-phase CC (5 × 30 cm) with gradient system from 33% to 100% MeOH to give eleven subfractions. F.5.3.8 was subjected to semi-prep HPLC with a MeOH/H_2_O (v/v, 66/34), resulting in the isolation of ginsenoside F_3_ (12.5 mg), 20(*S*)-ginsenoside Rg_2_ (32.1 mg), and 20(*R*)-ginsenoside Rg_2_ (21.2 mg). F.5.3.11 was subjected to semi-prep HPLC with a MeOH/H_2_O (v/v, 72/28 to 80/20), resulting in the isolation of 20(*E*)-ginsenoside F_4_ (21.0 mg). F.5.6 was subjected to Sephadex-20 (3 × 30 cm) with 100% MeOH to obtain six subfractions (F.5.6.1 – F5.6.6). ginsenoside Rh_18_ (10.5 mg) was purified from F5.6.5 using semi-prep HPLC with a MeOH/H_2_O (v/v, 59/41). F.5.6.6 was subjected to semi-prep HPLC with a MeCN/H_2_O (v/v, 23/77), resulting in the isolation of ginsenoside Re_4_ (28.6 mg). F.5.7 was subjected to reversed-phase CC (5 × 30 cm) with gradient system from 33% to 100% MeOH, resulting in the isolation of ginsenoside Re (111.6 mg) and ginsenoside Rc (5.0 mg).

F.6 (100 g) was subjected to seilica gel CC with solvent system of CHCl_3_ and MeOH to afford nine subfractions (F.6.1 – F.6.9). F.6.5 (3 g) was subjected to reversed-phase MPLC (5 × 20 cm) with gradient system from 25% to 100% MeOH to give five subfractions. F.6.5.2 was subjected to semi-prep HPLC with a MeOH/H_2_O (v/v, 55/45), resulting in the isolation of 20(*S*)-ginsenoside Rg_1_ (25.3 mg). F.6.6 (3.1 g) was subjected to reversed-phase CC (6 × 50 cm) with gradient system from 25% to 100% MeOH to give twenty-three subfractions. F.6.6.23 was subjected to semi-prep HPLC with a MeOH/H_2_O (v/v, 80/20 to 85/15), resulting in the isolation of notoginsenoside Fe (33.0 mg). F.6.7 was subjected to reversed-phase CC with gradient system of MeOH/H_2_O to give twelve subfractions. Vinaginsenoside R4 (75.6 mg) was isolated from F.6.7.12 by semi-prep HPLC with a MeOH/H_2_O (v/v, 68/20). F.6.8 was subjected to reversed-phase CC with gradient system of MeOH/H_2_O to give nineteen subfractions. F.6.8.10 was subjected to semi-prep HPLC with a MeOH/H_2_O (v/v, 66/34), resulting in the isolation of bipinnatifidusoside F_1_ (80 mg). F.6.8.18 was subjected to semi-prep HPLC with a MeOH/H_2_O (v/v, 80/20), resulting in the isolation of gypenoside XVII (70.4 mg). F.6.8.19 was subjected to semi-prep HPLC with a MeOH/H_2_O (v/v, 80/20), resulting in the isolation of gypenoside IX (49.7 mg). F.6.9 was subjected to reversed-phase CC (5 × 60 cm) with gradient system from 25% to 100% MeOH to give eighteen subfractions. F.6.9.18 was subjected to semi-prep HPLC with a MeOH/H_2_O (v/v, 66/34 to 70/30), resulting in the isolation of ginsenoside Rb_1_ (10 mg), ginsenoside Rb_2_ (38.2 mg), and ginsenoside Rb_3_ (16 mg).

Meanwhile, the methanol fraction which was kept for sapogenin production was subjected to the alkaline hydrolysis with the slight modification of the previous reports (Im *et al*., 1995; Cui *et al*., 1994). Final reacted fraction was subjected to silica gel CC with solvent system of *n*-hexane and EtOAc from 7:1 to 1:5 to obtain five subfractions (F.1′ – F.5′). F.2′ was subjected to semi-prep HPLC with a MeCN/H_2_O (v/v, 50/50 to 100/0), resulting in the isolation of dehydroprotopanaxatriol I (2.0 mg) and dehydroprotopanaxatriol II (3.1 mg). F.4′ was subjected to semi-prep HPLC with a MeOH/H_2_O (v/v, 93/7), resulting in the isolation of 20(*S*)-protopanaxadiol (11.3 mg) and 20(*R*)-protopanaxadiol (10.8 mg) were isolated by semi-prep HPLC with a MeOH/H_2_O (v/v, 95/5).

### Reactions to enhance the content of 20(*R*)-ginsenoside Rh_2_

In order to obtain 20(*R*)-ginsenoside Rh_2_ with the highest amount, reaction conditions were optimized as followed. Saponins rich fraction mentioned above was dissolved in D.W. to make 10% solution (w/v), to which viscozyme^®^, one of *β*-glucosidase, was added and subjected to reaction in a shaking incubator at 50 °C for 5 days. After incubation, resultant solution was autoclaved two times at 120 °C for 2 h each. The solution was subjected to LC/MS analysis for content evaluations (Fig. S7 and Table S2).

### WST-1 cell proliferation assay

After drug treatment, 10 µL of WST-1 reagent (Roche, Switzerland) was added to the cells, followed by incubation for 2 h. The culture plate was gently shaken for 1 min before absorbance was read at 450 nm using a microplate reader.

### Bromodeoxyuridine (BrdU) staining

Cells were seeded in 6 well plates at a density of 1.5 × 10^5^ cells/well. CPP531 was added 24 h later. The cells were treated with 10 µM BrdU 24 h before staining, After BrdU incubation, the media was discarded, the cells fixed by incubation with 10% formaldehyde for 15 min, and then permeabilized with 0.5 % Triton X-100 for 10 min. DNA was denatured by treatment with 2 N HCl for 10 min at 37 °C, followed by neutralization with 0.1 M boric acid pH 8.5 for 5 min at room temperature. Blocking was carried out using 5% bovine serum albumin (BSA) in PBST (0.2% TWEEN 20) for 1 h at RT. Cells were incubated with 1:500 BrdU antibody in 1% BSA diluted in PBST for 1 h at 37 °C, followed by washing with 1X PBS. Cells were then incubated with 1:1000 488 Alexa dye-conjugated anti mouse IgG (Thermo Fisher Scientific) in 1% BSA diluted in PBST for 1 hour at 37 °C, followed by washing with 1X PBS. The stained cells were mounted with Fluoromount™ solution including DAPI (Sigma-Aldrich)). BrdU staining was detected using an inverted fluorescence microscope (Leica DMI3000 B, Wetzlar, Germany).

### Western blotting

Cells were lysed with the CelLytic™ reagent (Sigma-Aldrich) and protein was quantified using the Bradford reagent (Bio-Rad, Hercules, USA). 50 µg protein samples were loaded onto 10% polyacrylamide gels, and transferred to nitrocellulose membranes after electrophoresis. Expression signals were detected with an ECL solution (RPN2232, GE Healthcare). Band intensity was quantified with ImageJ 1.45 s software (National Institutes of Health, Bethesda, USA).

### RT-PCR analysis

Cell were seeded in 6 well culture plates at a density of 1.5 × 10^5^ cells/well for compound treatment. RNA was isolated using the TRI-Solution™ (Thermo Fisher Scientific), following the manufacturer’s instructions. RT-PCR was performed using the AccuPower® RT PreMix (Bioneer, Daejeon, Korea). 1 µg of mRNA was used for the template. The primer sequences used were as follows: p27 forward 5′-GAG TCA GCG CAA GTG GAA TTT C-3′ reverse 5′-GCG AAG AAG AAT CTT CTG CAG C-3′ and p57 forward 5′-GCC GGT CGA GGA GCA GAA TG-3′ reverse 5′-CCT GGA GGG ACG TCG TTC GA-3′ and GAPDH forward 5′-TGA TGA CAT CAA GAA GGT GAA G-3′ reverse 5′-TCC TTG GAG GCC ATG TAG GCC AT-3′.

### Flow cytometric assessment of cell cycle status

C2C12 myoblasts were seeded in 60 mm × 15 mm culture dishes at a density of 2 × 10^4^ cells/well. 24 h later, myoblasts were treated with CPP531 for 72 h. Myoblasts were then harvested using trypsin-EDTA and stained with 2 μg/mL propidium iodide for 40 min. Flow cytometry was carried out using phycoerythrin (575 nm) detection in a BD FACSCanto II flow cytometer (Becton Dickinson, East Rutherford, USA).

### Assessment of cardiomyocyte proliferation in zebrafish

Wild type zebrafish were purchased from Lotte Mart, Gwangju, Korea, and maintained following standard protocols^68^. Embryos from mated *Tg(cmlc2:GFP)* transgenic zebrafish were grown in distilled water supplemented with 0.06 g/L sea salt. At 20 hours post fertilization (hpf), embryos were treated with 5 µg/mL CPP531 or vehicle until 40 hpf. After treatment, the compound solution was exchanged to egg water and the zebrafish were maintained until 72 hpf. For staining with 5-ethynyl-2′-deoxyuridine (EdU), zebrafish were treated with 10 mM EdU with 1% DMSO in 1X E3 buffer for 1 h at 28.5 °C, using a fish incubator. The fish were euthanized and fixed in 4% paraformaldehyde for 2 h. Fish larvae were then washed 3 times with PBDT buffer (0.1% TWEEN 20, 1% DMSO in PBS), followed by dehydration in 100% methanol for 1 h at −20 °C. Larvae were then sequentially rehydrated in a graded methanol series: 75%, 50%, 25% for 20 min each, with washing in PBDT for 20 min. The larvae were then digested with 10 µg/mL proteinase K or 20 min. After three further washes with PBDT, larvae were re-fixed in 4% paraformaldehyde for 20 min, followed by washing with PBDT and incubation with 12. 2 N HCl for 1 h at RT, 3 washes with PBDT, and incubation with 10% normal goat serum in PBDT for 1 h. EdU staining was carried out using a EdU imaging kit (Invitrogen, catalogue number C10340, Thermo Fisher Scientific). Larvae were then incubated with anti-EdU IgG in blocking serum overnight at 4 °C, washed three times with PBDT and incubated in goat anti-mouse IgG conjugated to Alexa-fluor 594 for 5 h in the dark. After washing with PBDT, the stained larvae were mounted and imaged using confocal microscopy (Olympus FluoViewTM FV1000, Tokyo, Japan).

### Animal model care and experimentation

Animal studies were carried out in accordance with guidelines established by the Animal Care and Ethics Committees of the Gwangju Institute of Science and Technology. The studies were approved by the Gwangju Institute of Science and Technology Animal Care and Use Committee (study approval number GIST-2015-07). Adult male 8-week-old Sprague Dawley rats (weight range 230–250 g) and 8-week-old male C57BL/6 mice (weight range 23–25 g) were used for the myocardial infarction model and muscle degeneration model, respectively (supplied by Damool Science, Daejeon, Korea). Animals were housed at a constant temperature of 28 °C with a 12 h/12 h light/dark cycle. Food and water were available *ad libitum* before surgery preparation and during drug treatment. Surgeries were performed under general anesthesia induced by intraperitoneal injection of 70 mg/kg ketamine (Yuhan, Seoul, Korea) and 15 mg/kg xylazine (Bayer AG, Leverkusen, Germany) dissolved in saline. CPP531 was dissolved in saline containing 20% PEG400 and 0.2% TWEEN 80. PEG400 is a low-molecular-weight grade of polyethylene glycol that possesses low toxicity and TWEEN 80 is an excipient that is used to stabilize aqueous formulations of drugs for injection into recipient animals. Animals were treated with 2 mg/kg CPP531 delivered by intraperitoneal (IP) injection every 24 h. The untreated control animals were treated with IP injection of vehicle alone. The injection volume was 1 mL for the rats and 100 µL for the mice.

### Myocardial infarction model

Myocardial infarction (MI) was induced in the rats by ligation of the anterior descending coronary artery, as previously described^69^. A 4-gauge polyethylene catheter was used for intubation and ventilated with room air using a rodent ventilator (Harvard Apparatus, Holliston, USA). The coronary artery was ligated with a 5-0 silk suture and 4-0 sutures were used to close the chest. The rats were then placed on a warm pad in the animal facility recovery room. CPP531 treatment commenced 24 h after surgery and animals were sacrificed after 7d.

### Echocardiography

Cardiac functional indices were obtained using echocardiography (Toshiba Power Vision 6000, Tokyo, Japan). Before taking echocardiographs, anaesthetized rats were left undisturbed for 5 min to stabilize the heartbeat. The following cardiac parameters were obtained: IVSTD (interventricular septal end diastole), LVIDd (left ventricular internal dimension, diastole), LVIDs (left ventricular internal dimension, systole), EF (ejection fraction), FS (fractional shortening) and ESV (end systolic volume).

### Skeletal muscle degeneration model

Intramuscular injection of barium chloride was used to induce muscle fiber degeneration, as previously described^28^. The right gastrocnemius muscle of anaesthetized mice was injected with 1.5% barium chloride dissolved in 50 µL saline using a 30-gauge syringe. Mice were transferred to a warm pad until they regained consciousness. After 7d CPP531 or vehicle treatment, the forced swimming capacity test was used to assess muscle function, as previously described^70^, with the following modifications: a glass beaker was used in place of an acrylic tank and a steel washer was not require to increase exercise burden. Animals were then sacrificed and the gastrocnemius muscle harvested. In a separate experiment, mice were treated with CPP531 or vehicle alone for 14 d, after which the animals were sacrificed and the gastrocnemius muscle harvested.

### Tissue harvesting and histological analysis

Dissected hearts were sliced horizontally in half, embedded in the OCT compound (Leica), and frozen at −80 °C. 1 d after freezing, cryosections were taken at a 7 µm thickness (Leica CM1850). Masson’s trichrome staining was performed using the previously published protocol^71^. Microscopic images were obtained at 25× magnification and the thickness of the left ventricle was measured using Image J 1.48 v software (National Institutes of Health).

To quantify proliferating cardiomyocytes in the heart tissues, immunofluorescence was performed as previously described^72^. In brief, the sections were immersed in acetone for 15 min, washed twice with PBS, incubated in 0.1% triton-X solution for 10 min and washed twice with PBS. Sections were then placed in 1 X citrate buffer and boiled for 40 sec in a microwave oven. Blocking was performed by incubation with 5% BSA (Merck, 821006, Kenilworth, USA) and 5% normal goat serum (Vector lab S-1000, Burlingame, USA) overnight at 4 °C. Sections were then incubated with primary antibody (anti-cardiac troponin T; Abcam ab8295, UK) and Ki-67 (CST D3B5, Cell Signaling Technology) diluted at 1:100 with 0.05% triton-X, overnight at 4 °C. Sections were then incubated with secondary antibody solution (goat anti-rabbit IgG-heavy and light chain antibody DyLight® 488 conjugated (Bethyl lab A120-101D2, Montgomery, USA)) and goat anti-mouse IgG-heavy and light chain antibody DyLight® 594 conjugated (Bethyl lab A 90-116D4) diluted 1:200 in 1X PBS) at 37 °C for 1 h. Sections were then treated for 30 min with sudan black B solution, immersed twice in a 1X MHB solution for 15 min, and stained with DAPI for 3 min. Fluorescence images were acquired at random by fluorescence microscopy (Leica DMI 3000B).

Dissected gastrocnemius muscles were frozen in isopentane pre-cooled by insertion into liquid nitrogen, embedded in OCT compound and stored at −80 °C. 1 d after freezing, 7 µm cryosections were taken and the sections stained with Masson’s trichrome or hematoxylin and eosin using a kit (Sigma-Aldrich). Microscopic images were obtained at 25x magnification to assess tissue degeneration and repair (Leica DM 2500). The ratio of damaged area to whole muscle tissue was obtained by analyzing Masson’s trichrome stained muscles using Image J 1.48 v software. The degenerated area was designated as purple staining and the non-damaged area was designated as red staining. To calculate the inflammatory score, 5 micrographs were taken at random from H&E stained sections of each treated muscle at 100x magnification (Leica DM 2500). Inflammatory cells were counted as lightly-stained cells with dark purple nuclei, which had infiltrated the muscle tissue between the darker red-stained muscle fibers. To analyze muscle fiber diameter, 5 images of the H&E stained muscles were selected at random using 200x magnification. Entire muscle fiber diameters present within the micrograph were measured with the Image J 1.48 v software.

### Statistical analysis

The Student’s *t*-test was used to determine statistical significance in Figs. 2D, 3B,D,F,G, 4A,E, 6A, C-E, and 6G–I. (Excel 2013, Microsoft Corporation, USA). One-way ANOVA was used to determine statistical significance in Figs. 1A,C, 2B, 3I,J, 4C, 5B and 5D, and two-way ANOVA was used to determine statistical significance in Fig. 1D (ANOVA was carried out using the Data Analysis ToolPak for Microsoft Excel 2013). A *p* value < 0.05 was considered to be significant. Unless otherwise stated, all cell-based data are generated from three independent experimental repeats and the error bars for the graphs are standard deviation.

## Supplementary information

Supplementary information.

## Data Availability

D.-W.J., D.R.W. and W.K.O. guarantee data accessibility in regard to this manuscript.
