# Supplementary material for: Screening ginseng saponins in progenitor cells identifies 20(R)-ginsenoside Rh2 as an enhancer of skeletal and cardiac muscle regeneration
Source: Sci Rep. 2020 Mar 18;10:4967. doi: 10.1038/s41598-020-61491-4 (PMC7080739; doi:10.1038/s41598-020-61491-4)
Supplement: Supplementary file 1 — Supplementary information. [file 41598_2020_61491_MOESM1_ESM.pdf]

Supplementary Information:

Screening ginseng saponins in progenitor cells identifies 20(R)-ginsenoside Rh_2 as an enhancer of skeletal and cardiac muscle regeneration

Ah Ra Kim[1,5#], Seon-Wook Kim[1#], Ba Wool Lee[2#], Kuk-Hwa Kim[2], Woong Hee Kim[1], Hong Seok[1], Ji-Hyung Lee[1], JungIn Um[1], Soon-Ho Yim[3], Youngkeun Ahn[4], Suk-Won Jin[5,6], Da-Woon Jung[1]*, Won Keun Oh[2]* and Darren R. Williams[1]*

[1]New Drug Targets Laboratory, School of Life Sciences, Gwangju Institute of Science and Technology, Gwangju, Jeollanam-do, 61005, Republic of Korea

[2]Korea Bioactive Natural Material Bank, Research Institute of Pharmaceutical Sciences, College of Pharmacy, Seoul National University, Seoul, Gyeonggi-do, 08826, Republic of Korea

[3]Department of Pharmaceutical Engineering, Dongshin University, Naju, Jeollanam-do, 58245, Republic of Korea

[4]Cell Regeneration Research Center, Department of Cardiology, Chonnam National University Hospital/Chonnam National University Medical School, Gwangju 61469, Republic of Korea

[5]Developmental Genetics Laboratory, School of Life Sciences, Gwangju Institute of Science and Technology, Gwangju, Jeollanam-do, 61005, Republic of Korea

[6]Yale Cardiovascular Research Center, Department of Internal Medicine, Yale University School of Medicine, New Haven, CT, 06511, USA

*Correspondence: 1) wkoh1@snu.ac.kr 2) darren@gist.ac.kr, 3) jung@gist.ac.kr.

[#]Equal contribution

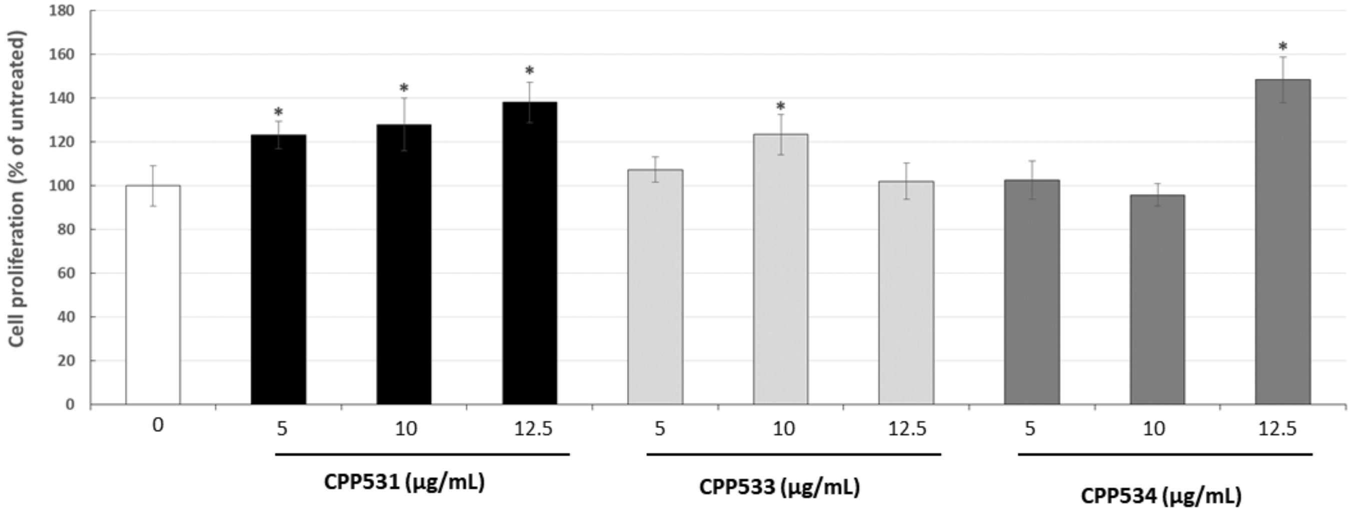

Figure S1 related to Figure 1: Effect of 20(R)-ginsenoside Rh_2 (CPP531), ginsenoside Rk_2 (CPP533) and isoginsenoside Rh_3 (CPP534) on C2C12 myoblast proliferation. *=p<0.05 and for significantly increased proliferation compared to the untreated cells (vehicle alone).

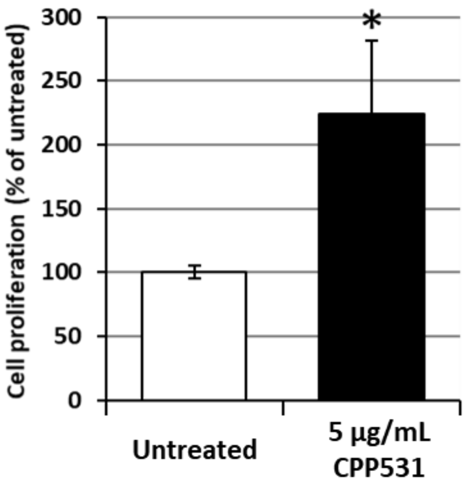

Figure S2 related to Figure 1: Effect of CPP531 on C2C12 proliferation as measured using the WST-1 assay. *=$p<0.05$ for significantly increased proliferation compared to the untreated cells (vehicle alone).

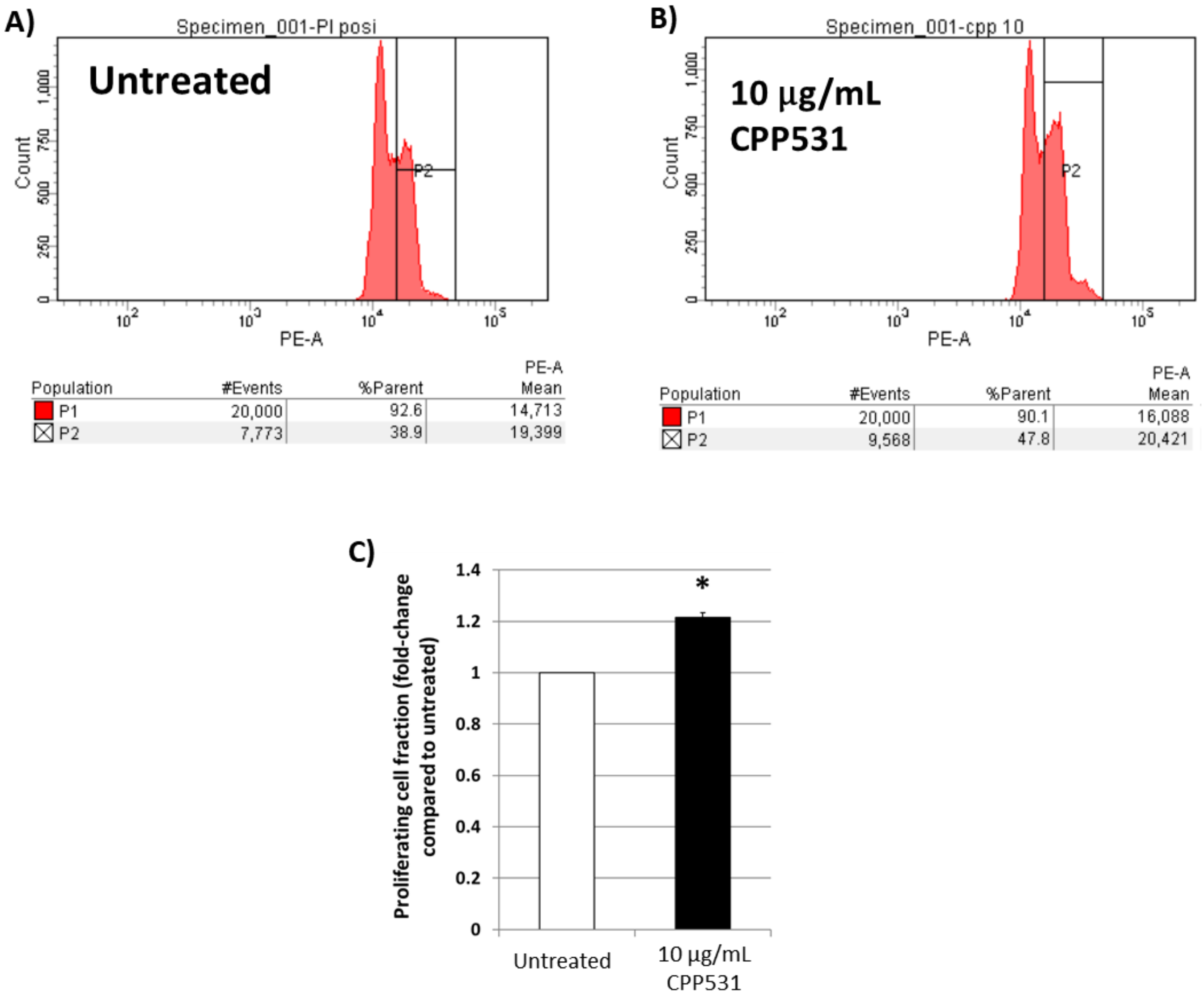

Figure S3 related to Figure 1: (A-B) Flow cytometric analysis of the effect of CPP531 treatment on cell cycle status in C2C12 myoblasts. Cells were treated with 10 µg/mL CPP531 for 72 h. (C) Quantification of the proliferating cell fractions (designated as ‘P2” in the flow cytometry histograms). *=p<0.05 for significantly increased proliferation compared to the untreated cells (vehicle alone).

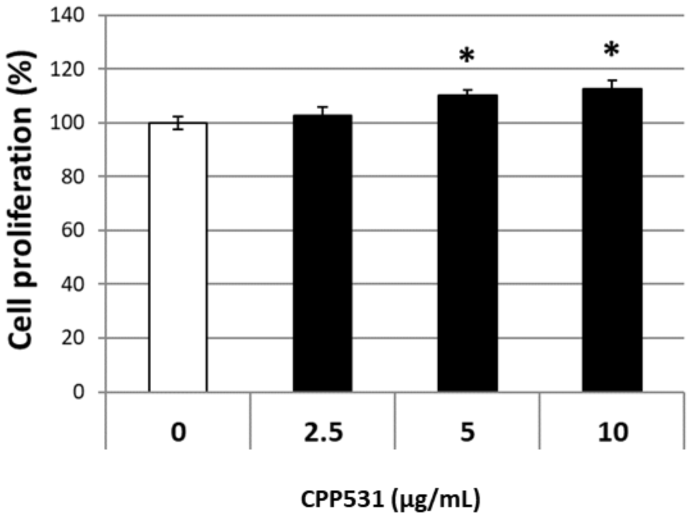

Figure S4 related to Figure 2: Dose-dependent effect of CPP531 treatment on human primary myoblast proliferation. The MTT assay was used to measure proliferation. *=p<0.05 for significantly increased compared to the untreated cells.

A)

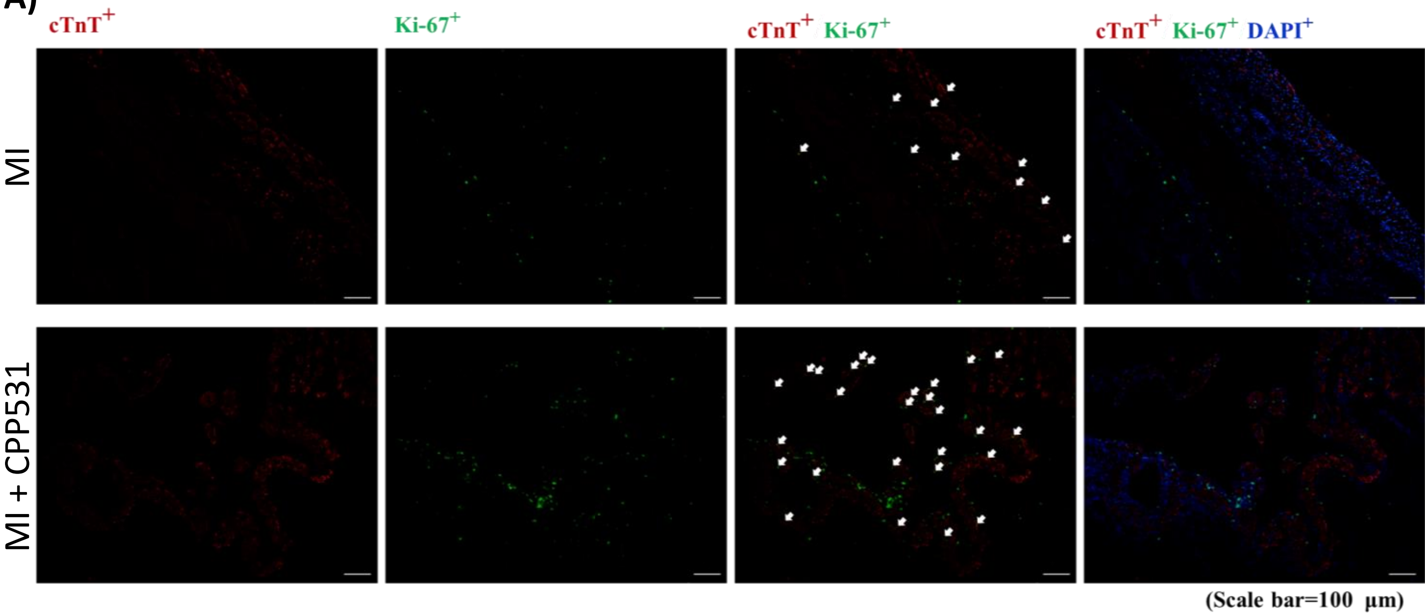

B)

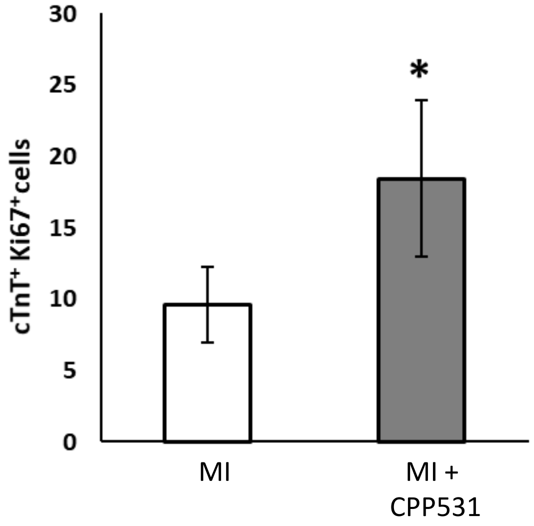

Figure S5 related to Figure 5: Immunostaining for proliferating cardiomyocytes in the infarcted left ventricle after 7 days treatment with vehicle alone or CPP531 (A) and quantification of proliferating cardiomyocytes (B). Cardiomyocytes were designated as Ki-67^{+} cells located within cardiac troponin T (cTnT) positive stained areas (shown using white arrows in (A)). *=p<0.05 for significantly increased proliferating cardiomyocytes.

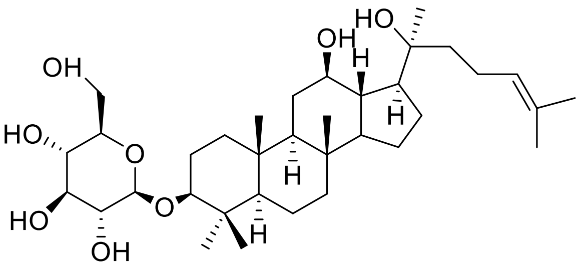

Figure S6 related to Figure 1: Structure of the non-hit ginsenoside, 20(S)-ginsenoside Rh_2 (CPP532).

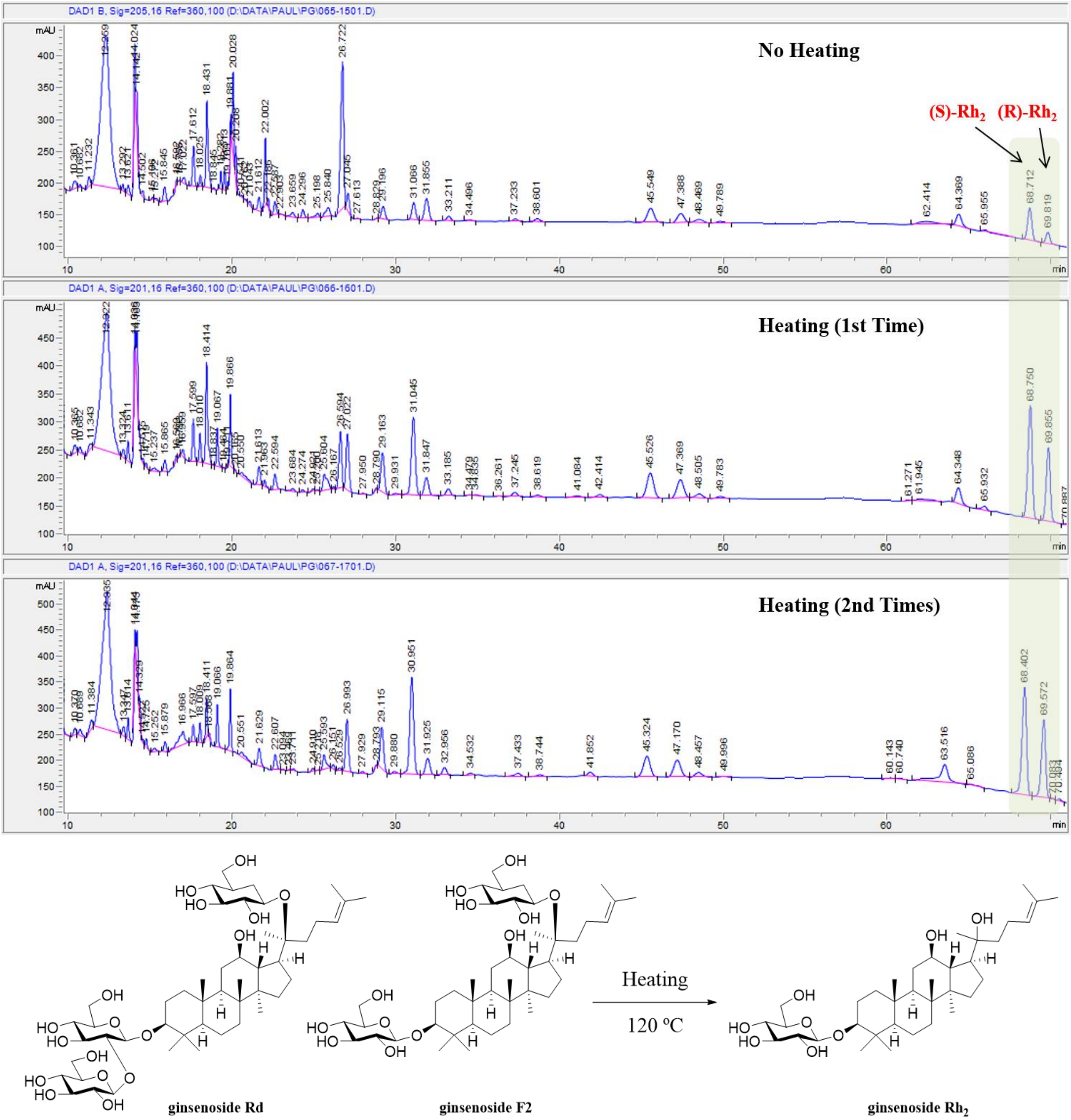

Figure S7 related to the Methods section: Chromatograms of ginseng extract after a series of reactions including enzyme and thermal hydrolysis.

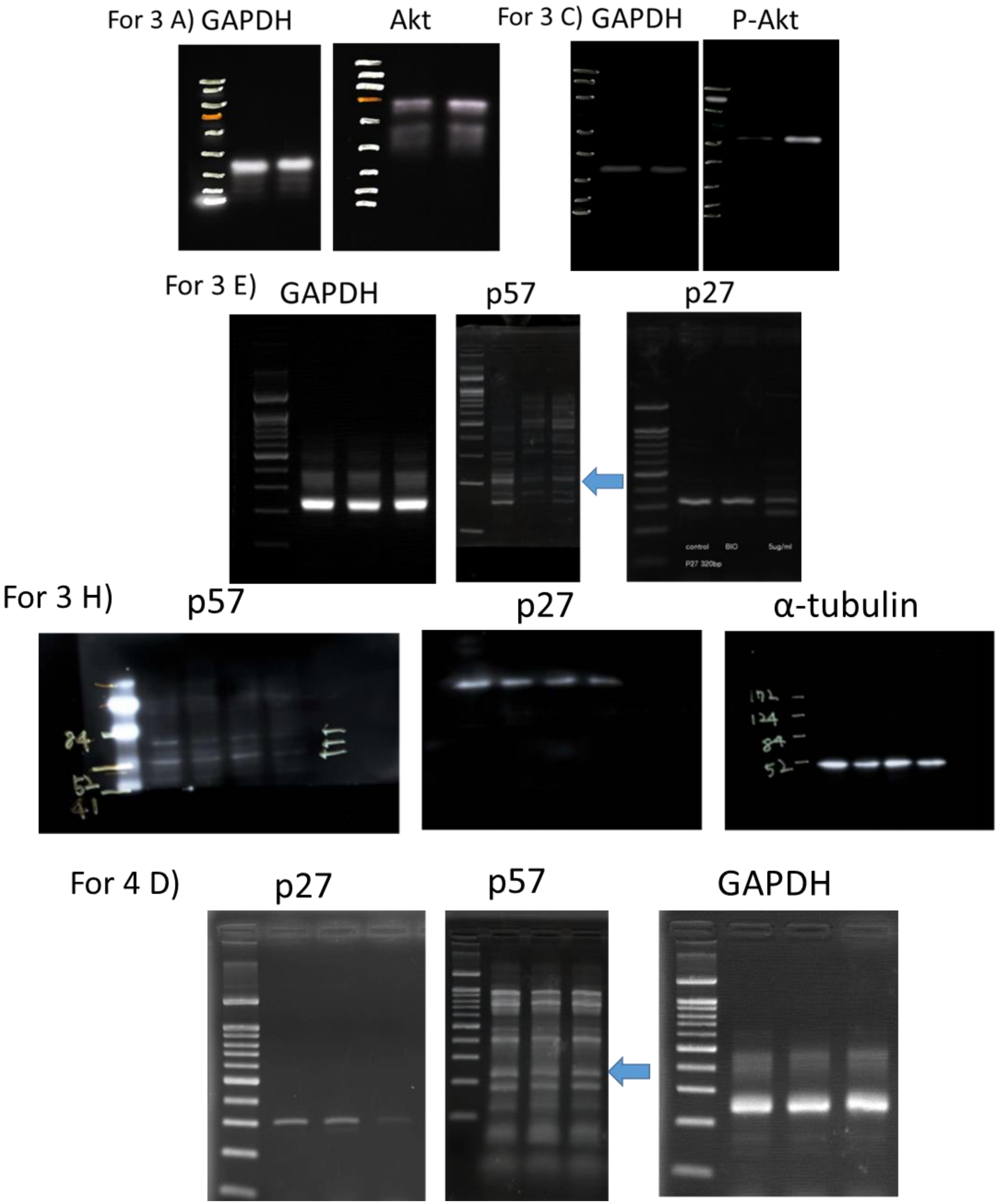

Figure S8 related to Figures 3A, 3C, 3E, 3H and 4D: Original blots for Figures 3A, 3C and 3H. Original gel scans for the RT-PCR data in Figures 3E and 4D. For the p57 RT-PCR results, a blue arrow designates the predicted PCR product size.

Table S1 related to the Methods section: ^{1}H and ^{13}C NMR Data of active compounds, 20(*R*)-ginsenoside Rh_2, ginsenoside Rk_2 and isoginsenoside Rh_3.

[a] spectra obtained at 500 MHz in pyridine-d_5.
[b] spectra obtained at 400 MHz in pyridine-d_5.

No.	20(R)-ginsenoside Rh_2[a]		ginsenoside Rk_2[a]		isoginsenoside Rh_3[b]	
	δ_H, mult. (*J* in Hz)	δ_C	δ_H, mult. (*J* in Hz)	δ_C	δ_H, mult. (*J* in Hz)	δ_C
1		39.6		39.7		39.6
2		27.2		27.2		28.5
3	3.40, dd (5.0, 15.0)	89.2	3.41, dd (3.5, 11.5)	89.2	3.39, dd (4.4, 12.0)	89.1
4		40.5		40.1		40.6
5	0.76, d (12.0)	56.8	0.77, d (10.0)	56.8	0.76, d (12.0)	56.8
6	1.47, m	18.9		18.9		18.8
7		35.6		35.8		35.7
8		37.4		40.6		40.0
9		50.8		51.3		51.1
10		40.1		37.5		37.4
11		32.6		33.1		32.6
12	3.94, m	71.3	3.92. m<br>2.10, m	72.8	3.93, m	72.9
13		49.7		52.9		50.8
14		52.2		51.6		51.4
15	2.03, m<br>1.08, m	31.9		33.0	1.93, m	33.0
16		27.1		31.2		27.1
17	2.39, m	51.1	2.84, m	48.7	2.00, m	51.3
18	1.03, s	17.8	1.03, s	16.2	1.03, s	16.8
19	0.84, s	16.8	0.83, s	16.9	0.83, s	17.1
20		73.4		155.9		140.5
21	1.41, s	23.1	5.17, br s<br>4.93, br s	108.5	1.83, s	13.5
22	1.74, m	43.7		34.3	5.52, t (7.6)	123.5
23	2.55, m<br>2.24, m	23.2	2.40, m<br>2.33, m	27.5	2.81, m	27.8
24	5.34, t (5.0)	126.5	5.31, t-like (5.5)	125.7	5.24, t (6.2)	124.2
25		131.2		131.6		131.6
26	1.74, s	26.3	1.67, s	26.2	1.64, s	26.0
27	1.67, s	18.2	1.61, s	18.2	1.60, s	18.1
28	1.01, s	16.3	1.34, s	28.6	1.33, s	29.2
29	1.34, s	28.6	1.02, s	17.2	1.02, s	16.2
30	1.03, s	17.2	0.99, s	17.4	0.99, s	17.4
1'	4.97, d (7.5)	107.4	4.98, d (7.9)	107.4	4.96, d (7.6)	107.3
2'	4.05, m	76.3	4.09, m	76.2	4.06, m	76.2
3'	4.28, m	79.2	4.28, m	79.2	4.27, m	79.1
4'	4.25, m	72.3	4.24, m	72.3	4.23, m	72.3
5'	3.95, m	78.8	4.04, m	78.8	4.03, m	78.7
6'	4.62, d (10.0)<br>4.42, dd (5.0, 10.0)	63.5	4.63, d (10.0)<br>4.43, d (5.0, 10.0)	63.5	4.61, dd (6.0, 12.0)<br>4.42, dd (6.0, 12.0)	63.5

Table S2 related to the Methods section: Content evaluation of ginsenoside Rh_2 from each stages of reactions using LC/MS.

*Auc : Autoclaved

Content evaluation	20(S)-ginsenoside Rh_2			20(R)-ginsenoside Rh_2		
	Area (mAU× min)	Content (μg)	Content (%)	Area (mAU× min)	Content (μg)	Content (%)
No reaction	-	-	-	-	-	-
Before Auc (pH 3)	1286.1	1.4528	1.4528	444.6	0.5204	0.5204
1st Auc (pH 3)	3716.7	4.2738	4.2738	2305	2.4834	2.4834
2nd Auc (pH 3)	3992.9	4.5944	4.5944	2701.9	2.9022	2.9022
3rd Auc (pH3)	3206.9	3.6821	3.6821	2258.2	2.4340	2.4340
